# Lectin Fingerprinting Distinguishes Antibody Neutralization in SARS-CoV-2

**DOI:** 10.1021/acscentsci.2c01471

**Published:** 2023-05-10

**Authors:** Michael G. Wuo, Amanda E. Dugan, Melanie Halim, Blake M. Hauser, Jared Feldman, Timothy M. Caradonna, Shuting Zhang, Lauren E. Pepi, Caroline Atyeo, Stephanie Fischinger, Galit Alter, Wilfredo F. Garcia-Beltran, Parastoo Azadi, Deb Hung, Aaron G. Schmidt, Laura L. Kiessling

**Affiliations:** † Department of Chemistry, Massachusetts Institute of Technology, Cambridge, Massachusetts 02139, United States; ‡ Ragon Institute of MGH, MIT, and Harvard, Cambridge, Massachusetts 02139, United States; § Department of Microbiology, Harvard Medical School, Boston, Massachusetts 02115, United States; ∥ The Broad Institute of MIT and Harvard, Cambridge, Massachusetts 02142, United States; ⊥ Koch Institute for Integrative Cancer Research, MIT, Cambridge, Massachusetts 02139, United States; # Department of Molecular Biology and Center for Computational and Integrative Biology, Massachusetts General Hospital, Boston, Massachusetts 02139, United States; ∇ Department of Genetics, Harvard Medical School, Boston, Massachusetts 02115, United States; ○ Complex Carbohydrate Research Center, University of Georgia, Athens, Georgia 30602, United States

## Abstract

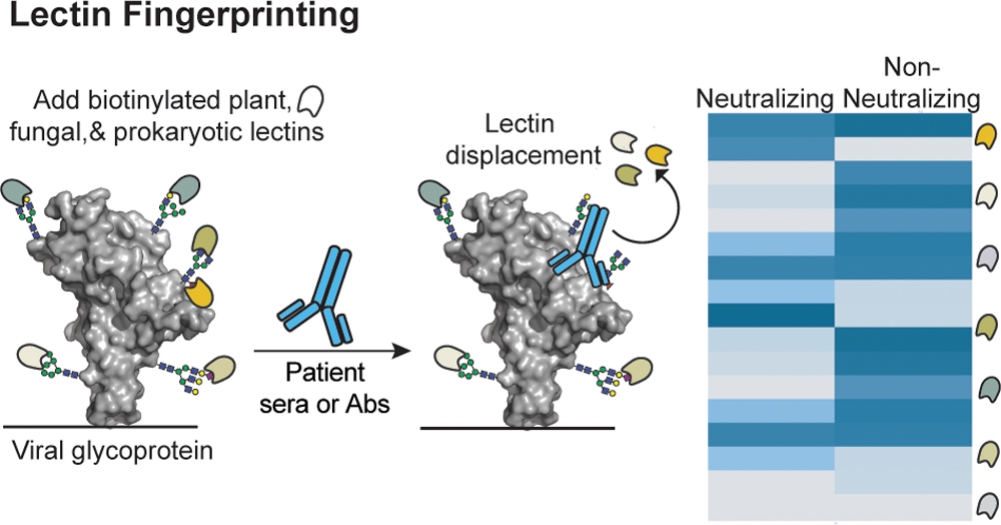

Enveloped viruses co-opt host glycosylation pathways to decorate their surface proteins. As viruses evolve, emerging strains can modify their glycosylation patterns to influence host interactions and subvert immune recognition. Still, changes in viral glycosylation or their impact on antibody protection cannot be predicted from genomic sequences alone. Using the highly glycosylated SARS-CoV-2 Spike protein as a model system, we present a lectin fingerprinting method that rapidly reports on changes in variant glycosylation state, which are linked to antibody neutralization. In the presence of antibodies or convalescent and vaccinated patient sera, unique lectin fingerprints emerge that distinguish neutralizing versus non-neutralizing antibodies. This information could not be inferred from direct binding interactions between antibodies and the Spike receptor-binding domain (RBD) binding data alone. Comparative glycoproteomics of the Spike RBD of wild-type (Wuhan-Hu-1) and Delta (B.1.617.2) variants reveal O-glycosylation differences as a key determinant of immune recognition differences. These data underscore the interplay between viral glycosylation and immune recognition and reveal lectin fingerprinting to be a rapid, sensitive, and high-throughput assay to distinguish the neutralization potential of antibodies that target critical viral glycoproteins.

## Abstract 2

Lectin fingerprinting is a high-throughput and facile method to rapidly profile the neutralization potential of patient antibodies raised against viral glycoproteins.

## Introduction

Viruses are the primary cause of respiratory tract infections worldwide, accounting for more than 2 million deaths annually.^1^ Viral pathogens have been responsible for some of the most significant infectious disease outbreaks recorded (poliovirus, 1918 influenza A, 1957 Influenza A; HIV/AIDS; SARS-CoV-2). Many viral pathogens share a common strategy of employing densely glycosylated surface proteins to facilitate viral entry into host cells and modulate host immune defenses.^2^ Because of their abundance and accessibility, viral glycoproteins often serve as key antigens upon which the host immune system mounts an attack. As the virus evolves, acquired mutations within these glycoproteins, such as the addition^3^ or remodeling of glycans,^4,5^ help the virus evade immune detection. Viral genome sequencing readily reveals amino acid mutations in emerging viral strains, yet alterations to protein glycosylation cannot be predicted from sequencing information alone. New tools to rapidly profile changes in viral protein glycosylation and their impact on immune recognition would be valuable assets in monitoring viral pathogens.^6^

**Figure 1:**
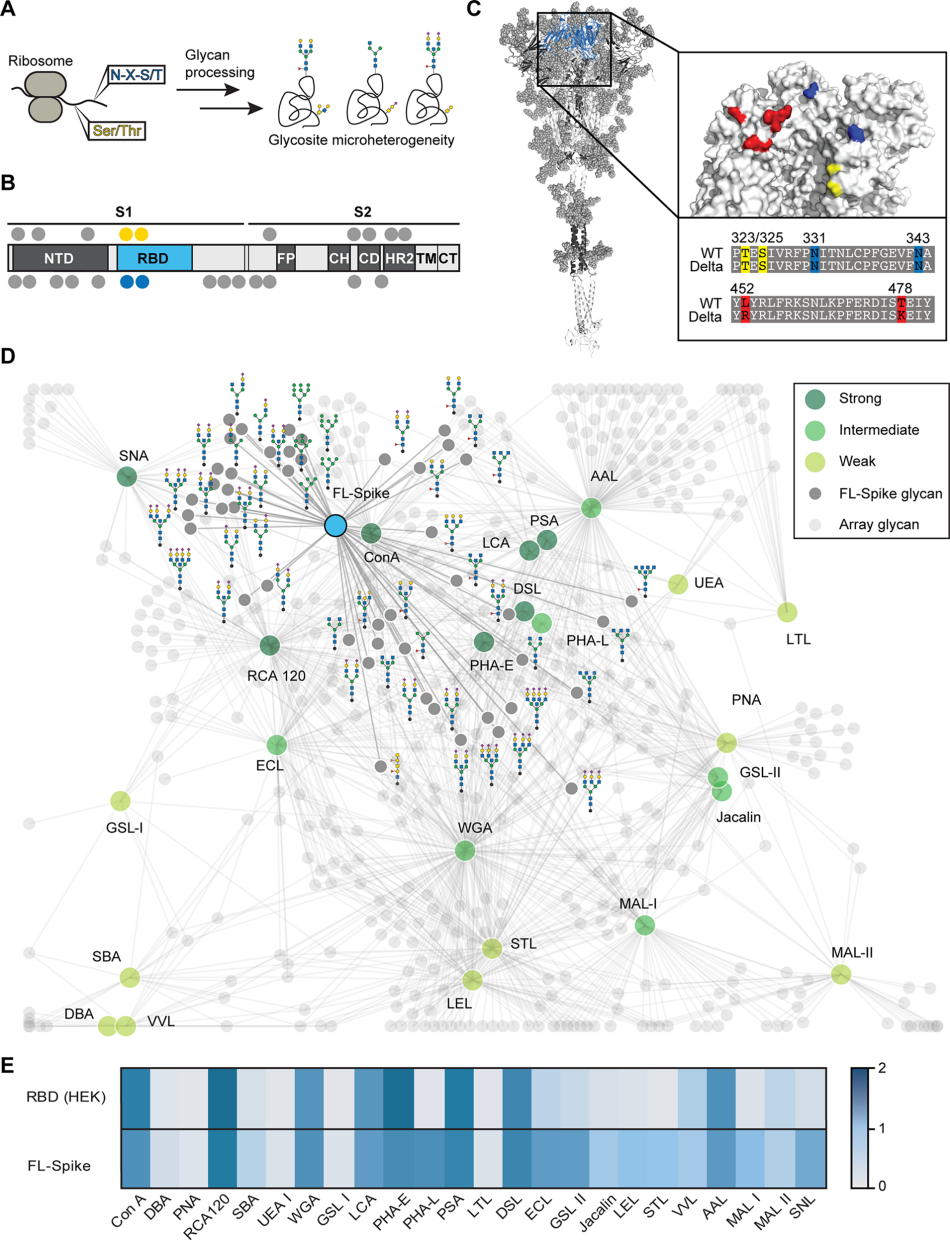
Glycosylation sites mapped onto the SARS-CoV-2 Spike glycoprotein. (A) Glycan processing following protein translation leads to sequence microheterogeneity at N- and O-glycosites (N-X-S/T and S/T, respectively) where X is any amino acid except proline. (B) The multidomain WT and Delta Spike glycoproteins contain the receptor binding domain (RBD, blue). Predictions suggest 22 N-glycans (gray, blue for RBD) and two O-glycans (yellow) are present in each monomer of the trimeric Spike glycoprotein. (C) Sequence alignment of WT and Delta Spike RBD region showing S323/T325 (yellow), N331/343 (blue), and Delta mutations L452R and T478K (red). (D) Interaction map of 46 distinct glycans present on FL-Spike and 24 plant and fungal lectins. Predicted strong (dark green), intermediate (green), and weak (light green) binders have decreasing number of shared glycans. (E) Lectin fingerprints for RBD and FL-Spike are shown.

Human coronaviruses are emblematic of viruses that undergo mutation, but the corresponding changes in protein glycosylation tend to be enigmatic. The coronavirus SARS-CoV-2 uses its Spike glycoprotein, which harbors significant glycosite microheterogeneity^7−9^ (Figure 1A), to mediate contacts with host receptors and facilitate entry into cells.^10^ Spike glycoprotein is a multidomain, class I fusion protein that exists as a homotrimer on the virion surface; the Spike protein is densely glycosylated, containing two putative O-glycosylation and 22 predicted N-glycosylation sites per monomer in the wild type (Wuhan-Hu-1) strain (Figure 1b).^8,9^ Although viral entry is primarily mediated by interactions between the receptor-binding domain (RBD) of the Spike glycoprotein and host angiotensin-converting enzyme 2 (ACE2) receptor, recent studies have demonstrated that Spike glycans enhance viral entry through interactions with host lectins.^11,12^ Thus, modification of N- and O-glycans on Spike could influence host responses to SARS-CoV-2.

Changes in viral glycosylation pose a risk to global vaccination efforts. With SARS-CoV-2, the host humoral response is predominantly directed against the Spike protein;^5,13,14^ still, not all antibodies that bind Spike can neutralize the virus and prevent viral entry into host cells.^15,16^ Moreover, variants with changes proximal to Spike glycosites have emerged during the pandemic.^17−19^ These evolved strains are of growing concern as they exhibit heightened transmissibility and may reduce the neutralization efficacy of convalescent or vaccinated patient antibodies.^3,20−22^

Two types of assays are traditionally used to determine the neutralization capacity of antibodies to SARS-CoV-2: (1) cellular assays that measure the ability of antibodies to block viral infection or (2) in vitro assays that screen for the ability of antibodies to directly compete with Ace2 receptor binding. The former requires specialized equipment/facilities and is low throughput, and the latter assay does not account for antibodies that bind Spike at positions distal to the Ace2 recognition site but are nonetheless capable of neutralizing the virus. As such, complementary platforms that rapidly screen patient antibodies for Spike-neutralizing or non-neutralizing capabilities would be advantageous for understanding the spread of SARS-CoV-2 and informing therapeutic and vaccine design.

The ability of enveloped viruses to alter their glycan coat to evade antibody neutralization is well-documented. Spike is the major glycoprotein present on coronavirus envelopes, and antibody recognition occurs on epitopes proximal to or in direct contact with known glycosites.^23−25^ We reasoned that tools to quickly and easily report on changes in Spike glycosylation among viral variants would reveal insights into how viruses such as SARS-CoV-2 modify their glycans to evade immune surveillance. Moreover, these glycan reporters could be leveraged to generate recognition patterns, or fingerprints, from neutralizing and non-neutralizing antibodies. Unique fingerprints generated by antibodies with distinct mechanisms of action (e.g., direct competition with receptor and coreceptor binding sites) could then serve as standards for evaluating if patient sera contain antibodies with similar properties. Here we report a lectin fingerprinting strategy to predict the neutralization potential of antibodies against WT and Delta SARS-CoV-2 variants and understand how viruses utilize glycosylation to subvert host immune defenses.

## Results

### Lectin Toolkit to Map SARS-CoV-2 Spike Glycosylation

The Spike glycoprotein of SARS-CoV-2 contains overlapping yet distinct glycan compositions at each of its many glycosylation sites.^8,9^ Given advances in glycan profiling and diagnostics,^26−28^ we reasoned that nonantibody carbohydrate-binding proteins, or lectins, could detect changes in viral glycan microheterogeneity. Because glycans abound on the Spike glycoprotein and its RBD, we reasoned that lectins would bind differentially and compete with neutralizing antibodies. Thus, we set out to identify a collection of lectins to fingerprint the Spike RBD.

We began by using the Glycan Array Dashboard (GLAD) tool^29^ to collate the deposited glycan array data sets for 24 plant- and fungal-derived lectins against 611 glycans.^30^ Lectins displaying overlapping specificities and those containing distinct carbohydrate recognition properties were identified using correlation analysis (Figure S1) and publicly available data.^31^ The glycan-binding specificities of each lectin were subsequently mapped onto the 46 unique glycoforms reported for each of the 74 binding sites on the trimeric ectodomain of recombinant full-length Spike (FL-Spike) to predict which lectins would recognize Spike glycans (Figure 1D). From our glycan interaction mapping, we expected 14 lectins to bind with intermediate to strong affinities and 10 to interact minimally with FL-Spike.

To test the in silico projections, we optimized an enzyme-linked lectin assay (ELLA) that would allow us to identify which lectins from the curated suite bind FL-Spike and act as reporters of glycosylation. We immobilized HEK-expressed FL-Spike via passive adsorption to a hydrophilic 96-well plate. Biotinylated lectins were applied to the Spike protein-coated wells, and lectins bound to FL-Spike glycans were detected with StrepTactin-HRP conjugate (Figure S2). FL-Spike has limited amounts of exposed galactose, *N*-acetylgalactosamine (GalNAc), and fucose (Fuc). Lectins recognizing these carbohydrates [*Dolichos biflorus* agglutinin (DBA, α-GalNAc), peanut agglutinin (PNA, Gal (β 1,3) GalNAc), *Ulex europaeus* agglutinin I (UEA I, α-Fuc, arabinose), *Griffonia simplicifolia* lectin I (GSLI, α-Gal), and *Lotus tetragonolobus* lectin (LTL, α-Fuc, arabinose)] showed minimal binding to FL-Spike.

Of the 24 plant and fungal lectins tested, 19 bound FL-Spike with a moderate to strong signal. Lectin binding was dose-dependent (Figure S3), indicating that complexation is specific. Additionally, treating FL-Spike with the N-glycosidase PNGase F reduced lectin binding, confirming that protein–glycan interactions drive the observed signal (Figure S4). Several lectins predicted to bind weakly [*Lycopersicon esculentum* lectin (LEL, specificity for GlcNAc), *Solanum tuberosum* lectin (STL, GlcNAc), *Vicia villosa* lectin (VVL, GalNAc), *Maackia amurensis* lectin II (MALII, α-2,3-sialic acid), and soybean agglutinin (SBA, Gal, GalNAc)] still gave moderate signals. The variance between predicted and experimental data may arise from changes in viral glycan display and minor differences in recombinant protein preparation. Our data indicate that lectin valency also alters Spike recognition. For example, AAL, UEA I, and LTL can all interact with fucose residues, but AAL is the best Spike binder. AAL is a dimer with five fucose binding sites per monomer (10 sites total). In contrast, UEA I is a dimer of monomers with one binding site each (2 sites total), and LTL is a tetramer of monomers with one binding site each (4 sites total). Thus, AAL, the lectin predicted to have the most multivalent interactions, exhibited the highest functional affinity.

### Lectin Fingerprints Differentially Report on SARS-CoV-2 Domains

We next evaluated the generality of lectin fingerprinting and its ability to report on glycosylation of different SARS-CoV-2 domains. We posited that the RBD of Spike would generate a lectin fingerprint distinct from the FL-Spike. As anticipated, the RBD bound fewer lectins than FL-Spike (Figure 1E). To determine how expression cell line affected glycosylation of Spike domains, we compared the lectin fingerprint of recombinant RBD from HEK and insect cells and found distinct lectin binding signatures, in agreement with recently published reports (Figure S5).^32^ These data indicate that the lectin binding is specific and can rapidly report on differences in glycosylation for SARS-CoV-2 glycoproteins.

### Neutralizing Antibodies Compete with Lectins for SARS-CoV-2 Spike

**Figure 2:**
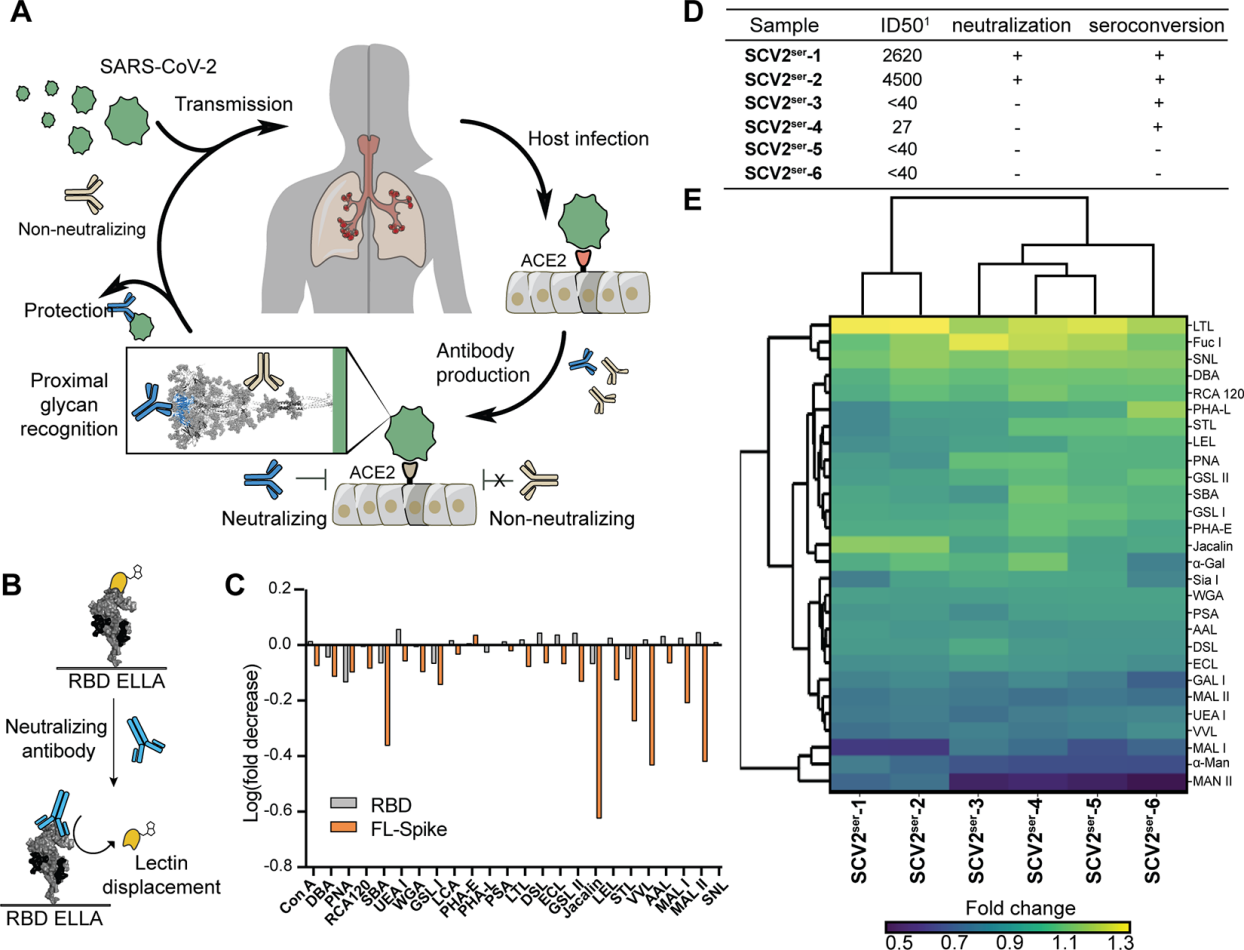
Lectin fingerprints reveal neutralizing potential of prevaccinated patient antibodies and patient-derived sera. (A) Host infection by SARS-CoV-2 elicits a rapid humoral response producing either neutralizing or non-neutralizing antibodies. (B) Competitive ELLA assay to reveal unique lectin fingerprints. (C) Log (fold change) upon treatment of immobilized RBD (gray) and FL-Spike (blue) containing 24 plant and fungal lectins with MM43 commercial anti-RBD antibody (1 μg/mL) in parallel. (D) Patient sera metadata table used in blinded study (*n* = 8) indicating seropositivity and estimated RBD abundance. Serum dilution in pseudovirus inhibition assay performed with pseudotyped virus indicates competitive ID_50_ values. (E) Dendrogram displaying hierarchical clustering analysis of immobilized RBD with bound plant, fungal, and prokaryotic lectins (1 μg/mL) treated with convalescent patient sera (1:100 dilution). Distinct and separate lectin fingerprints were observed for each sample class. Dendrograms from neutralizing serum (rows 1 and 2, *n* = 2) and non-neutralizing (rows 3–6, *n* = 4). Hierarchical analysis represents data from two separate experiments.

Upon infection with SARS-CoV-2, patients rapidly develop a humoral response to viral antigens (Figure 2A). In many cases, the antibodies generated in response to SARS-CoV-2 infection are protective. Most characterized neutralizing antibodies engage the RBD, and many interact with the ACE2 orthosteric binding site.^33^ Proximal to the ACE2 binding site are two N-glycans, N331 and N343, and the corresponding glycans in the MERS-CoV and SARS-CoV-1 Spike protein contact neutralizing antibodies. We therefore postulated that neutralizing antibodies would compete with lectins for Spike occupancy, leading to competitive displacement of lectins upon the addition of neutralizing antibody.

Antibodies themselves carry a single N-glycosylation mark that could complicate the interpretation of the lectin fingerprints.^34^ We initially examined lectin binding to immobilized commercial antibodies (1 μg/mL) and found that, with the exception of ConA and LCA, minimal interactions were detected relative to those obtained using RBD binding (Figure S6). These data indicate that antibody–RBD interactions, and not antibody glycans themselves, should be responsible for competitive fingerprints obtained upon antibody addition.

To test if antibodies compete with lectins that bind Spike glycans, we evaluated an S1 neutralizing antibody raised against Val16-Arg685 of recombinant Spike (αS1-MM43). The antibody’s ability to displace lectins bound to immobilized RBD was assessed in a competitive ELLA assay (Figure 2B). Competitive lectin fingerprints for the RBD differed when antibodies were present (Figure S7A). A separate competitive fingerprint was generated for FL-Spike. Displacement patterns for the lectins jacalin (Gal(B1,3)GalNAc, O glycan), SBA (GalNAc, Gal), and STL (GlcNAc) were observed for both Spike and the RBD (Figure S7B). A direct comparison (Figure 2C) showed a greater decrease in lectin binding when antibodies were added to FL-Spike. Taken together, these findings indicate that the assay is sensitive to the number of glycosites disrupted upon antibody binding.^35^

The data led us to ask whether a panel of lectins might distinguish neutralizing from non-neutralizing antibodies. We therefore queried whether neutralizing and non-neutralizing antibodies purified from convalescent patients would possess unique lectin fingerprints. In addition to the 24 plant and fungal lectins, we introduced six commercially available prokaryotic lectins with defined monosaccharide specificities (recombinant prokaryotic lectins, or RPLs, Table S1) to expand the detection of glycan microheterogeneity. We characterized the lectin’s unique lectin fingerprints to FL-Spike and RBD (Figure S8).

To avoid complications from FL-Spike structural and conformational plasticity, we used the RBD for competitive lectin fingerprints from patient antibodies. We tested four monoclonal antibodies purified from convalescent patients (**SCV2**^**mAb**^**-1**–**SCV2**^**mAb**^**-4**) and two commercial mouse antibodies that were cross-reactive with SARS-CoV-1 RBD but were either known or likely to be non-neutralizing against SARS-CoV-2 (**α-RBD-D002** and **α-S1-CR3022**). To reduce the high dimensionality of competitive lectin fingerprints, we employed hierarchical clustering analysis with Euclidean distance measure,^36^ where the similarity between individual lectin data is calculated combinatorially. The row and column dendrograms are automatically generated with seriation to find similarities in the lectin fingerprints.^37^ Application of this protocol indicated that commercial neutralizing antibodies cluster separately from patient antibodies, indicating differences in lectin displacement. Within the patient antibody cluster, neutralizing antibody **SCV2**^**mAb**^**-1** grouped separately from the other patient antibodies. Weakly neutralizing antibody **SCV2**^**mAb**^**-2** and neutralizing antibody **SCV2**^**mAb**^**-3** clustered together, indicating a possible shared mechanism of binding that is distinct from **SCV2**^**mAb**^**-1**. The non-neutralizing antibody, **SCV2**^**mAb**^**-4**, clusters broadly with patient antibodies when compared with commercial antibodies, but separately from the other neutralizing patient antibodies (Figure S7C). As indicated by the hierarchical clustering analysis, the profiles of α-S1 cross-reactive SARS-CoV-1 antibodies (**α-RBD-D002** and **α-S1-CR3022**), which engage RBD distal to the ACE2 binding site, are comparable.^38^ These results suggest that lectin fingerprinting can distinguish unique or overlapping binding modes of patient antibodies to Spike RBD.

### Convalescent Patient Sera Display Distinct Competitive Lectin Fingerprints

Rapid serological tests^39^ to evaluate the neutralization capacity of patient antibodies would be valuable in evaluating antibody efficacy of vaccine formulations. We reasoned that prevaccination patient-derived sera rather than purified antibodies could be used to generate unique lectin fingerprints predictive of antibody protectiveness. To this end, we obtained blinded serum from six convalescent patients. Two patients possessed neutralizing antibodies against WT SARS-CoV-2 (**SCV2**^**ser**^**-1**, **SCV2**^**ser**^**-2**), two had antibodies that bound WT Spike RBD but were non-neutralizing by an in vitro infection assay (**SCV2**^**ser**^**-3**, **SCV2**^**ser**^**-4**), and two generated antibodies that had no apparent Spike binding or neutralization activity (**SCV2**^**ser**^**-5**, **SCV2**^**ser**^**-6**) (Figure 2D). Competitive lectin fingerprints were subjected to hierarchical clustering analysis by Euclidean measure (Figure 2E) and by principal component analysis (PCA) (Figure S9). In each analysis, the clustering patterns separated by sera type. Both samples with neutralizing serum clustered together, while non-neutralizing convalescent sera showed distinct lectin fingerprints. These initial findings indicate that competitive lectin fingerprinting could be benchmarked and used as a diagnostic tool for evaluating sera neutralizing potential.

### O-Glycosylation Differences in Spike Drive Antibody Efficacy in Vaccinated Patients

**Figure 3:**
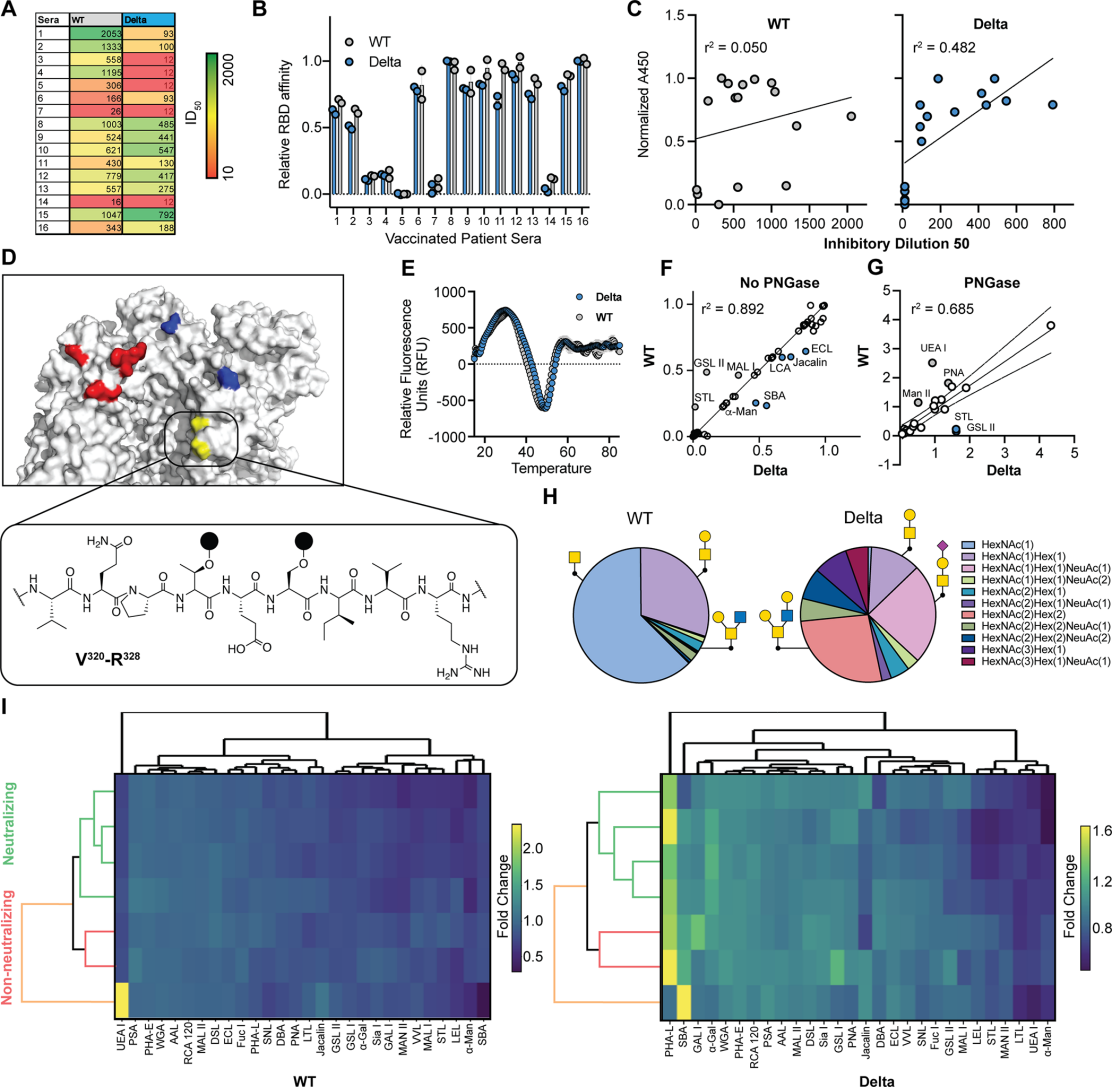
Relation of O-glycosylation differences of VOCs to antibody recognition in vaccinated patient sera. (A) Human patient sera (*n* = 16) and their neutralization potential (ID_50_) against WT and Delta VOC in a pseudovirus neutralization assay. (B) Patient sera binding to Spike RBD variants assessed by ELISA. (C) Correlation of neutralization data to normalized ELISA data of WT (left) and Delta (right). (D) O-Glycosite and trypsin digestion fragment analyzed by glycoproteomics (E) Differential scanning fluorimetry of WT and Delta Spike RBD. Lectin fingerprinting comparison of WT over Delta glycans with (F) no PNGase F and (G) PNGase F treatment. (H) Glycoproteomics data from WT (left) and Delta (right) Spike RBD showing the three most abundant glycans detected. (I) Dendrogram displaying hierarchical clustering analysis of immobilized WT (left) or Delta (right) Spike RBD with bound plant, fungal, and prokaryotic lectins (1 μg/mL) and vaccinated patient sera (BNT162b2, 1:100 dilution). Dendrograms from neutralizing serum (green, *n* = 4), non-neutralizing (red, *n* = 2), and intermediate (orange, *n* = 1). Hierarchical analysis represents data from two separate experiments

Robust neutralizing antibody responses against the WT and other variants of concern (VOCs) have been generated from mRNA (mRNA)-based vaccination against SARS-CoV-2 Spike protein. We analyzed sera collected from individuals that received either one or two doses of mRNA-1273 and BNT162b2 mRNA vaccines (Figure 3A, Table S2). Collected sera elicited ID_50_ values between 12 and 2053 in a SARS-CoV-2 infection assay with WT and Delta strains (Figure 3A). The observed variability in neutralization was not reflected in sera affinity for the WT and Delta Spike RBD, which were similar across all samples by ELISA (Figure 3B). We found no correlation (WT, *r*^2^ = 0.050; Delta, *r*^2^ = 0.482) between relative Spike RBD affinity and pseudovirus neutralization (Figure 3C).

Viruses are notorious for altering their amino acid sequences during evolution. The effects of these changes on neighboring glycosylation sites is poorly understood. The Delta variant has two amino acid changes in its Spike RBD, L452R and T478K (Figure 3D), that are proximal to glycosites S323/T325 and N331 and N343. We hypothesized that the changes in these residues could yield alterations in glycosylation that impact neutralization assays. The WT and Delta Spike variants had no differences in conformational stability as judged by differential scanning fluorimetry (Figure 3E). We next used lectin fingerprinting to examine changes in N- and O-glycosylation and identified that WT and Delta glycosylation are highly correlated (*r*^2^ = 0.892).

Lectins with differences in their binding to WT versus Delta include ECL (specificity for Gal, GalNAc, Lac), GSL II (GlcNAc), jacalin (sialylated T-antigen, Gal), LCA (Man, Glc), MAL I (Gal, Lac), SBA (Gal, GalNAc), STL (GlcNAc), and α-Man. These lectins recognize features of O-glycans suggesting that WT and Delta strains have major differences in O-glycosylation (Figure 3F). To confirm whether recognition was specific to O-glycans, we performed lectin fingerprinting after removing N-glycans and observed enrichment in the binding of GSL II, STL, UEA I, PNA, and Man II (Figure 3G). To determine if the lectin fingerprints were representative of direct O-glycan changes, we performed comparative glycoproteomics on WT and Delta Spike RBD (V320-R328). Although both variants contained GalNAc-Gal at this glycosite (WT, 30.2%; Delta, 12.0%), clear composition differences were obtained. The WT strain had mostly GalNAc at S323/T325 (62.3%) while Delta contained 24.1% GalNAc-Gal-NeuAc and 26.7% HexNAc(2)Hex(2), neither of which were observed in the WT variant. That SARS-CoV-2 alters its O-glycosylation to subvert host immunity is consistent with recent reports that the Omicron variant installs a novel O-glycosylation site to facilitate immune evasion.^19^

We next asked if lectin fingerprinting could assess the neutralizing potential of vaccinated patient sera by analyzing samples from patients who received the BNT162b2 mRNA vaccine (Figure 3I). Sera neutralization assays showed a broad distribution of neutralizing and non-neutralizing antibodies. For both WT and Delta RBD, we found that neutralizing and non-neutralizing sera exhibited lectin fingerprints that were distinct from one another, but highly overlapping between samples within a given group. These data suggest that, within a cohort vaccinated with the same antigen, neutralization can be predicted by lectin fingerprinting against the WT and Delta.

### Different mRNA Vaccines Yield Sera with Distinct Lectin Fingerprints

**Figure 4:**
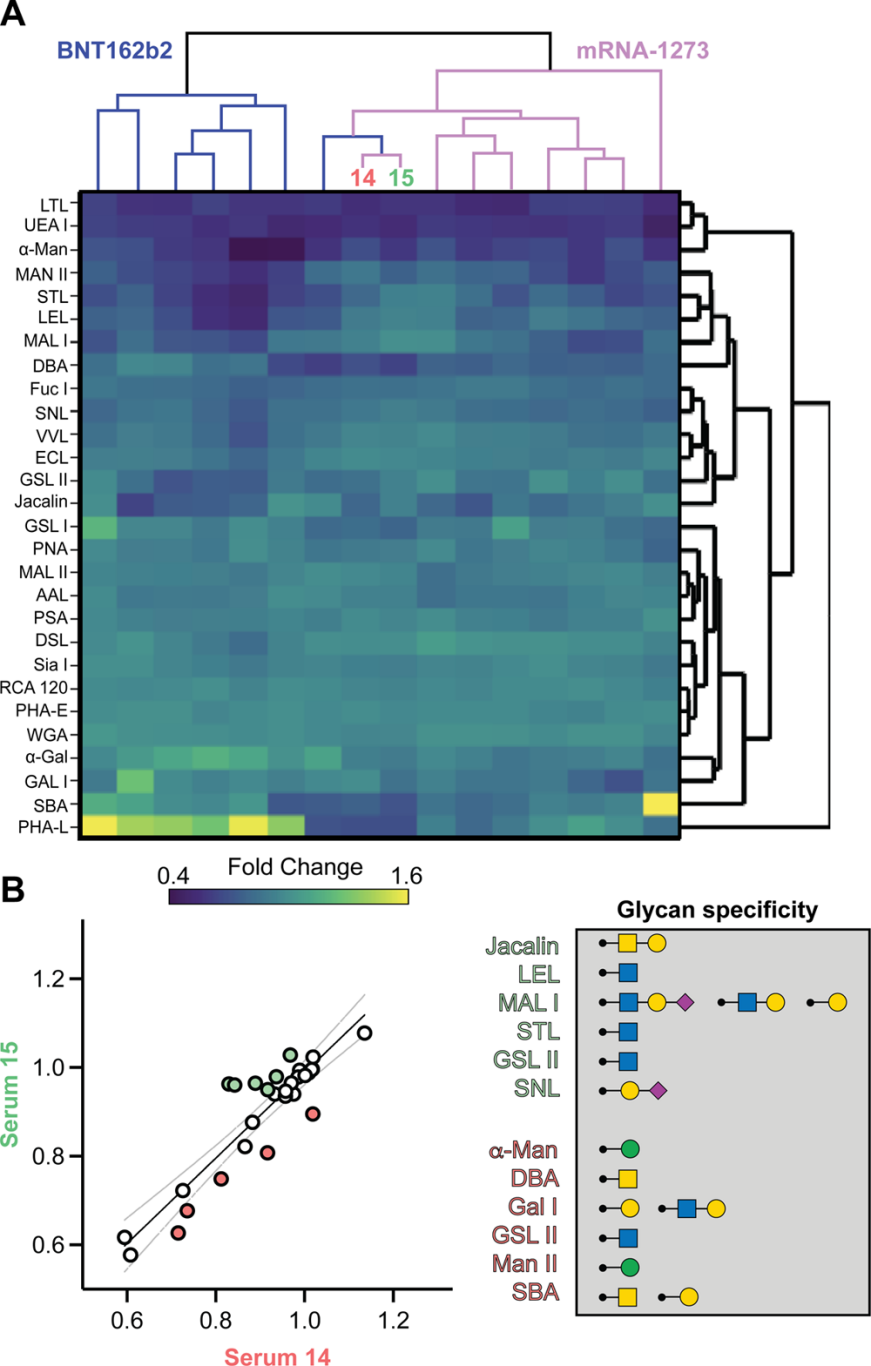
mRNA vaccine strategy yields distinct lectin fingerprints. (A) Dendrogram displaying hierarchical clustering analysis of immobilized Delta Spike RBD with bound plant, fungal, and prokaryotic lectins (1 μg/mL) treated with vaccinated patient sera BNT162b2 (purple, *n* = 7) or mRNA-1273 (pink, *n* = 9) diluted 1:100. Hierarchical analysis represents data from two separate experiments. (B) Correlation analysis of Serum 15 and Serum 14 (mRNA-1273) with enriched lectins shown in green and red, respectively (left). Glycan specificity of enriched lectins (right).

BNT162b2 and mRNA-1273 vaccination use different mRNA Spike sequences to elicit an antibody response.^40^ To determine whether mRNA vaccination strategy changes how vaccinated patient sera engage Spike RBD, we performed lectin fingerprinting on all patient sera (*n* = 16). Hierarchical clustering analysis of lectin fingerprinting showed dissimilar patterns from the BNT162b2 and mRNA-1273 vaccinees, suggesting that differential vaccination strategies elicit variations in Spike RBD glycoforms. The result is lectin fingerprints that vary with vaccination strategy (Figure 4A). Patients 14 and 15 received the mRNA-1273 vaccine, and serum from 15 could potently neutralize all VOCs tested whereas that from patient 14 did not neutralize any VOC (Table S1). We therefore conducted a lectin fingerprint correlation analysis (Figure 4B). Lectin fingerprinting revealed that glycan binding for serum 15 was enriched for lectins that engage GlcNAc and N-acetylneuraminic acid, whereas serum 14 enriched for galactose- and mannose-binding lectins. These data suggest that the broadly neutralizing serum 15 engages Spike RBD epitopes proximal to O-glycans while broadly non-neutralizing serum 14 engages protein epitopes between N- and O-glycans.

## Discussion

As SARS-CoV-2 variants emerge and evolve for immune escape, changes in glycosite composition as well as the appearance of new glycosites^3^ will inevitably impact antibody recognition. Few low-cost tools exist to rapidly profile protein glycosylation and report on glycoprotein–protein interactions (gPPIs). In the case of SARS-CoV-2 infections, where glycosylation of the Spike protein plays a central role in viral infection and immune evasion, such tools would illuminate how viral protein glycosylation changes over the course of infection and its inevitable evolution between hosts. In this study, we developed a lectin fingerprinting method that provides a snapshot of glycan microheterogeneity on recombinant FL-Spike and its RBD.

To generate this lectin fingerprint, we used 24 plant and fungal lectins and six prokaryotic lectins to index the accessible glycans on the viral protein surfaces. Lectin fingerprints for FL-Spike and RBD were consistent with mass spectrometric analysis of SARS-CoV-2 glycoproteins, demonstrating the accuracy of lectin fingerprinting as a readout of protein glycosylation. Additionally, lectin fingerprinting rapidly reported on differences in glycosylation for SARS-CoV-2 glycoproteins produced in different cell types. Together, the data suggest that this method may be useful for evaluating alterations to protein glycosylation during proteostatic stress (e.g., influenza infection) or in viral infections with multitissue tropism that could contribute to viral glycan heterogeneity, depending on the cell type propagating the virus.^27^ Unlike other methods for profiling protein glycosylation (e.g., MS analysis, lectin microarrays), lectin fingerprinting requires minimal sample processing and uses common laboratory equipment and reagents. Furthermore, this method can be readily scaled up for high-throughput screening applications and easily formatted for probing any glycoprotein of interest.

Although Still, not all antibodies generated against RBD are neutralizing, recent reports indicate that the majority of anti-SARS-CoV-2 neutralizing antibodies target the RBD. These observations suggest that privileged epitopes exist on RBD and targeting of these sites by a host may confer increased immunity against the virus. The assays currently used to examine binding of convalescent patient antibodies to RBD cannot distinguish which sites on RBD are bound, nor which antibodies are neutralizing. However, structural studies on antibody–Spike interactions suggest that antibodies make contacts with glycans on RBD. Taken together, we reasoned that tools capable of distinguishing antibody engagement of RBD could offer a new route to evaluate convalescent patient antibodies and their likelihood of conferring protection from SARS-CoV-2 infection. Such reagents could also uncover mechanisms of glycan-mediated immune evasion.

If anti-SARS-CoV-2 antibodies engage epitopes on Spike RBD proximal to its glycosites, we postulated that antibodies should be able to competitively displace bound lectins and perturb the lectin fingerprint. Indeed, we found that commercially available neutralizing antibodies bound proximally to exposed glycosites and perturbed the lectin fingerprints. Upon binding RBD, non-neutralizing antibodies also perturbed the lectin fingerprint, but in a manner that was distinct from neutralizing antibodies. These data indicate that, in engaging their respective epitopes, antibodies competitively displace lectins in a pattern that corresponds to their neutralization capacity. We employed lectin fingerprinting to evaluate the neutralization potential of antibodies within prevaccination convalescent patient sera. The use of patient sera is more relevant for clinical evaluations but demands the evaluation of antibodies within a more complex mixture of serum glycoproteins. We found that convalescent sera yielded distinct lectin fingerprints that corresponded to their neutralization efficacy.

Glycan changes have been implicated in stabilizing Spike protein structure and influencing immune selective pressure.^41,42^ Differences in S323/T325 O-glycosylation in the Spike RBD of WT and Delta variants highlight a potentially critical nexus in the evasion of SARS-CoV-2. When O-glycosylation was reduced, as seen in the mutation of T372A from scanned SARS-CoV-2 genomes, infectious virus showed increase replication^42^ suggesting that a delicate balance of glycosylation status is critical for maintaining immune evasion and infectivity. The differences in antibody responses elicited from BNT162b2 and mRNA-1273 that encode different Spike antigens result in changes in glycosylation. How population heterogeneity selects for specific glycan microheterogeneity may influence the evolution of SARS-CoV-2.

Our data suggest that lectin fingerprinting can be used as a diagnostic tool for evaluating antibody efficacy against SARS-CoV-2 antigens and other glycosylated proteins. We anticipate that the method can be used to profile alterations in SARS-CoV-2 glycosylation that are present in rapidly emerging, highly infectious VOCs. Understanding how changes in glycan structure influence antibody recognition may reveal new insights into how new viral variants are able to evolve to evade immune responses. More broadly, we believe that lectin fingerprinting can be used to map protein glycosylation for other proteins in which glycosylation plays a key functional role.

## Supporting Information Available

Materials and methods including detailed experimental procedures about protein production, ELLAs, neutralization assays, hierarchal clustering analysis, and comparative glycoproteomics (MS) analysis; detailed reagent information including antibodies, lectins, and sera used in these studies; additional figures showing plant and fungal monosaccharide specificity, schematics for ELLA and competitive ELLA assays, additional lectin binding data to Spike and RBD under varying treatment conditions, and lectin binding to commercial antibodies (PDF)
Tables sourcing the lectins used and vaccinated patient metadata (ZIP)
