# Supplementary material for: Lectin Fingerprinting Distinguishes Antibody Neutralization in SARS-CoV-2
Source: ACS Cent Sci. 2023 May 10;9(5):947–56. doi: 10.1021/acscentsci.2c01471 (PMC10214521; doi:10.1021/acscentsci.2c01471)
Supplement: Supplementary file 1 — oc2c01471_si_001.pdf [file oc2c01471_si_001.pdf]

Supplementary Materials for

Lectin fingerprinting to map protein glycosylation of SARS-CoV-2

Authors: Michael G. Wuo[1*], Amanda E. Dugan [1*], Melanie Halim[1], Blake M. Hauser [2], Jared Feldman[2], Timothy M. Caradonna [2], Shuting Zhang[4,6,7], Lauren E. Pepi [8], Caroline Atyeo [2], Stephanie Fischinger [2], Galit Alter [2], Wilfredo F. Garcia-Beltran[2], Parastoo Azadi[8], Deb Hung [4,7,7], Aaron G. Schmidt [2,3], Laura L. Kiessling[1,4,5]

*These authors contributed equally to this work

* To whom correspondence should be addressed: kiesslin@mit.edu

Affiliations:

1. Department of Chemistry, Massachusetts Institute of Technology, Cambridge, MA, USA
2. Ragon Institute of MGH, MIT, and Harvard, Cambridge, MA, USA.
3. Department of Microbiology, Harvard Medical School, Boston, MA, USA.
4. The Broad Institute of MIT and Harvard, Cambridge, Massachusetts, USA
5. Koch Institute for Integrative Cancer Research, MIT, Cambridge, Massachusetts, USA
6. Department of Molecular Biology and Center for Computational and Integrative Biology, Massachusetts General Hospital, Boston, Massachusetts, USA
7. Department of Genetics, Harvard Medical School, Boston, Massachusetts, USA
8. Complex Carbohydrate Research Center, University of Georgia, Athens, Georgia, USA

This PDF file includes:

Methods and Materials

Figs. S1 to S9

Tables S1 & S2

Materials and Methods

Reagents

Biotinylated plant and fungal lectins were purchased from Vector Labs. Lectins received as lyophilized powders were resuspended at 2 mg/mL in 20 mM HEPES, 150 mM sodium chloride. Biotinylated prokaryotic lectins were provided by GlycoSeLect, Ltd. (Dublin, Ireland) (**Table S1**). SARS-CoV-2 FL-Spike and RBD were recombinantly expressed as previously described.[1] Recombinant FL-Spike protein was stored in PBS at 4 °C for up to 1 week. Recombinant RBD was aliquoted and stored at –20°C.

Human subjects

A power analysis was performed to determine the number of vaccinated patient samples required for this study. We estimated effect size to be modest (0.3-0.5) to strong (0.8) based on the perturbation of lectins by commercial neutralizing antibodies. Using these data, we estimated that 4-16 patient samples would be required to achieve 80% power with a significance level alpha = 0.05.

Pre-vaccinated patient sera was collected in compliance with IRB protocol IRB2020P000849, Biorepository for samples from those at increased risk for or infected with SARS-CoV-2. Use of vaccinated patient sera for the development and validation of SARS-CoV-2 diagnostic tests was approved by Partners Institutional Review Board (IRB2020P000895). Serum samples from 16 patients diagnosed with COVID-19 (confirmed by at least one SARS-CoV-2 PCR-positive nasopharyngeal swab at Massachusetts General Hospital) were collected over course of several weeks, resulting in partially longitudinal, cross-sectional cohort consisting of 16 serum samples. Antibodies **α–S1-MM43** (Mouse mAb, 40591-MM43, Sino Biological, epitope Val16-Arg685 on the S1 portion of recombinant Spike protein), **α-RBD-D002** (Chimeric mAb,

40150-D002, Sino Biological, Arg306-Phe527 on recombinant Spike RBD) and **α-S1-CR3022** (ab273073-200UG, Abcam, binds the amino acids 318-510 in the S1 domain of the SARS-CoV Spike protein as well as SARS-CoV-2 (COVID-19) Spike protein) were purchased and stored according to manufacturers' recommendations. Patient antibody samples were collected in compliance with IRB protocol 2020P000895 and vaccinated patient sera were collected in compliance with IRB protocol 2020P002274.

Cell lines

HEK293T cells (ATCC) were cultured in DMEM (Corning) containing 10% fetal bovine serum (VWR), and penicillin/streptomycin (Corning) at 37°C and 5% CO_2. 293T-ACE2 cells were a gift from Michael Farzan (Scripps Florida) and Nir Hacohen (Broad Institute) and were cultured under the same conditions as HEK293T cells. Confirmation of ACE2 expression in 293T-ACE2 cells was done via flow cytometry. The African Green Monkey Vero E6 with stable TMPRSS2 expression was constructed by lentiviral transduction of Vero E6 (ATCC) using the construct pTRIP-SFFV-Hygro-2A-TMPRSS2 and selection at 500 µg/ml hygromycin. Vero E6 TMPRSS2 + cells were maintained in Dulbecco's Modified Eagle's Medium (DMEM, 25 mM glucose, 4 mM glutamine, 1 mM sodium pyruvate, Gibco, #11965118) supplemented with 10% fetal bovine serum (FBS), 100 U/mL penicillin/streptomycin and 250 µg/ml hygromycin at 37 °C with 5% CO_2.

SARS-CoV-2 Lentiviral neutralization assay

Neutralization was determined using a SARS-CoV-2 pseudovirus expressing a luciferase reporter gene, as described previously[2]. To generate the pseudovirus, the packaging construct psPAX2 (Cat# 11348, AIDS Reagent), luciferase reporter plasmid pLenti-CMV Puro-Luc (Cat# 17447, Addgene) and Spike protein expressing pcDNA3.1-SARS CoV-

2.SΔCT were transfected in HEK293T cells by the calcium phosphate method at a ratio of 1:1:0.5. Supernatants were collected and filtered with a 0.45-μm filter 48 hours post-transfection. For the neutralization assay, HEK293Ts were transfected with pcDNA3.1(-)-hACE2 (Cat# 1786, Addgene). The hACE2-expressing HEK293T cells were plated in 96-well plates 12 hours after transfection at a density of 20,000 cells/well and rested overnight. Serum was heat inactivated by incubation at 56 °C for 30 minutes. Heat inactivated serum was twofold serially diluted, mixed with 50 uL of pseudovirus, and incubated at 37 °C incubator for 1 hour. After incubation, the serum/pseudovirus mixed was added to the HEK293T/hACE2 cells. Six hours after infection, cell medium was replenished. Cells were lysed in Steady-Glo Luciferase Assay (Promega) 48 hours after infection. A luciferase assay was performed with luciferase assay reagent (Promega) according to the manufacturer's protocol. NT50 was defined as the concentration of serum required to achieve half maximal neutralization.[1]

Authentic SARS-CoV-2 neutralization assay

To evaluate neutralization activity of serum samples against authentic SARS-CoV-2, Vero E6-TMPRSS2 were seeded at 10,000 cells per well in CellCarrier-384 ultra microplate (Perkin Elmer) the day prior to infection. Patient serum samples were tested at a starting dilution of 1:40 and were serially diluted 4-fold up to four dilution spots. Serial diluted patient sera were mixed separately with diluted SARS-CoV-2 virus and incubated at 37 °C with 5% CO_2 for 1 hour. Sera-virus complexes were added to the cells. Plates were incubated at 37 °C with 5% CO_2 for 48 hours. Cells were fixed and inactivated using 4% paraformaldehyde in PBS for 2 hours at room temperature. Plates were then washed and incubated with diluted anti-SARS-CoV/SARS-CoV-2 nucleoprotein mouse antibody (Sino) for 1.5 hours at room temperature. Plates were

subsequently incubated with Alexa488-conjugated goat anti-mouse (JacksonImmuno) for 45 mins at room temperature, followed by nuclear staining with Hoechst 33342 (ThermoFisher). The fluorescence images were recorded and analyzed using Opera Phenix™ High Content Screening System. The half-maximal inhibitory dilutions (ID_{50}) were determined using four parameters nonlinear curve fitting algorithm.

Lectin Fingerprinting Enzyme Linked Lectin Assay (ELLA)

FL-Spike and RBD were diluted in PBS pH 7.5 to a final concentration of 10 μg/mL. 50 uL FL-Spike or RBD solution was plated onto 96 well Nunc MaxiSorp plates, sealed, and left to adsorb overnight at 4 °C . Plates were blocked with 5% BSA in PBS containing 0.1% Tween-20. Blocking occurred for 2 h, r.t. or overnight at 4 °C . Biotinylated lectins were diluted to 1 μg/mL in 20 mM HEPES pH 7.4, 150 mM sodium chloride, 10 mM calcium chloride, 0.1 % Tween-20, 0.1 % BSA. For dose dependence studies, lectins were added at 1, 10, 100 μg/mL. Lectins were added in duplicate (50 uL per well) and allowed to bind for 2 h, r.t., with agitation. Plates were washed three times with PBS containing 0.1 % Tween 20 (PBST). Lectins were visualized with StrepTactin-HRP conjugate (BioRad, cat. no. 1610381; 1:10,000 dilution in 20 mM HEPES pH 7.4, 150 mM NaCl, 10 mM CaCl2, 0.1 % BSA, 0.1 % Tween-20), 1 hour, r.t. with agitation. Plates were washed three times with PBST. Each well received 50 uL of 1-Step Ultra TMB-ELISA substrate solution (Thermo Fisher cat. 34028), and the reactions were quenched with 50 uL of 1 M sulfuric acid. Time of TMB-ELISA incubation was equal across wells, and wells were quenched in same order of addition. Plates were read at 450 nm. Assays were run in duplicate across experiments.

Competitive ELLA

FL-Spike, WT RBD, and Delta RBD were diluted in PBS pH 7.5 to a final concentration of 10 μg/mL. 50 uL FL-Spike or RBD solution was plated onto 96 well Nunc MaxiSorp plates, sealed, and left to adsorb overnight at 4 °C. Plates were blocked with 5% BSA in PBS containing 0.1% Tween-20. Blocking occurred for 2 h, r.t. or overnight at 4 °C. Biotinylated lectins were diluted to 1 μg/mL in 20 mM HEPES pH 7.4, 150 mM NaCl, 10 mM CaCl2, 0.1 % Tween-20, 0.1 % BSA. Lectins were added in duplicate (50 uL per well) and allowed to bind for 2 h, r.t., with agitation. Plates were washed three times with PBS containing 0.1 % Tween 20 (PBST). 50 uL of antibodies (1 μg/mL in 20 mM HEPES pH 7.4, 150 mM NaCl, 10 mM $CaCl_2$, 0.1 % BSA) or sera (1:100 dilution in 20 mM HEPES pH 7.4, 150 mM NaCl, 10 mM $CaCl_2$, 0.1 % BSA) were added to corresponding wells and allowed to bind for 1 hour, r.t., with agitation. Plates were washed three times with PBST. Lectins were visualized with StrepTactin-HRP conjugate (BioRad, cat. no. 1610381; 1:10,000 dilution in 20 mM HEPES pH 7.4, 150 mM NaCl, 10 mM CaCl2, 0.1 % BSA, 0.1 % Tween-20), 1 hour, r.t. with agitation. Plates were washed three times with PBST. Each well received 50 uL of 1-Step Ultra TMB-ELISA substrate solution (Thermo Fisher cat. 34028), and the reactions were quenched with 50 uL 1 M sulfuric acid. Time of TMB-ELISA incubation was equal across wells, and wells were quenched in same order of addition. Plates were read at 450 nm.

PNGase treatment

1 μg WT RBD, Delta RBD and FL-Spike were subjected to PNGase F (NEB, P0704S) treatment under native and denaturing conditions, according to the manufacturer's protocol. Briefly, 1 μg RBD and FL-Spike were each combined with 2 uL 10X glycobuffer, 2 uL 10% NP-40, and water to 20 uL. 2 uL of PNGase F was added and

incubated overnight at 37 °C to yield deglyosylated native proteins. For denaturing protocol, 1 μg RBD and FL-Spike were combined with 1 uL 10X denaturing glycobuffer and water up to 10 uL final volume. Proteins were denatured at 95 °C for 10 minutes and then cooled on ice briefly. 1 uL PNGase was added to the denatured proteins and incubated for 1 hour at 37 °C . Proteins were combined with gel loading buffer and run on SDS-PAGE (Bio-Rad, 4-15% Mini-Protean TGX Stain free gel, cat. 4568086). Proteins were visualized on Chemi-Doc MP system (Bio-Rad) after excitation with UV to activate stain-free reaction.

Comparative Glycoproteomics of WT and Delta Spike RBD

50 μg WT and Delta RBD were dissolved in 25 μL digestion buffer (50 mM Ammonium Bicarbonate) and homogenized. 25 μL of 25 mM dithiothreitol (DTT) was added and incubated at 45°C for 45 mins, followed by 25 μL of 90 mM iodoacetamide and incubated at room temp in the dark for 20 mins. Following incubation, samples were desalted using 10 kDa Amicon Ultra centrifuge filters (cat. UFC501096). The sample was then digested with Trypsin (1:20 E:S) at 37°C overnight. The reaction was stopped by heating samples to 100°C for 5 min. The sample was then de-N-glycosylated using 2 μL of PNGase F (NEB) and incubated at 37°C overnight. The samples were then loaded onto a C18 SPE cartridge (Resprep cat. 26030), and the released N-glycans were eluted with 5% acetic acid. The de-N-glycosylated peptides were then eluted with 20%, 40% and 100% IPA in 5% acetic acid, respectively. Eluted peptides were then dried down and reconstituted in 0.1% formic acid for LC-MS/MS analysis.

Glycopeptides were analyzed using an Orbitrap Eclipse Tribrid mass spectrometer equipped with an RSLCnano system. A prepacked nano-LC column (15 cm length, 75 μm ID and filled with 3 μm C18 material) was used. LC-MS/MS runs were conducted for

180 mins. Precursor ion scan was acquired at 120,000 resolution in the Orbitrap analyzer, and precursors at a time frame of 3 sec were selected for subsequent MS/MS fragmentation in the Orbitrap analyzer at 15,000 resolution. MS/MS fragmentation was conducted with stepped higher-energy collisional dissociation (HCD) product triggered collision induced dissociation (CID) (HCDpdCID). This method was set to trigger a CID experiment when oxonium ions indicative of glycosylation were seen in the HCD spectrum. Precursors with an unknown charge state, or charge state of +1 were excluded, and dynamic exclusion was enabled (30 sec duration). Glycopeptides were detected and assigned by Byonic software, Thermo Fisher Freestyle 1.8 and manual interpretation. Glycopeptides were searched against the FASTA sequence of the RBD region of the WT and Delta Spike proteins, and tryptic cleavage sites were searched (semispecific cleavage enabled). Oxidation of methionine, carbamidomethylation of cysteine and deamidation of asparagine and glutamine, as well as common human O-glycans were searched as variable modifications[3–5].

Data analysis

To determine the fold change of lectin binding upon sample treatment (antibody or serum condition), ratio of untreated lectin control signals to treated conditions were calculated. Untreated controls were run as internal standards for each experiment. Hierarchical clustering heatmaps were generated using heatmaply package in RStudio[6]. Within the heatmaply package, row dendrograms were automatically calculated using the hclust function with a Euclidean distance measure and average linkage function.

Glycan array data analysis and mapping

Publicly available glycan microarray data was obtained from Center for Functional Glycomics (CFG). Plant and fungal lectin microarray data was either processed at 1 μg/mL (AAL, Con A, DSL, GSL I, GSL II, Jacalin, LCA, LEL, PHA-E, PSA, RCA 120, SBA, SNA, STL, UEA I, VVL, WGA), 5 μg/mL (ECL), 10 μg/mL (DBA), or 100 μg/mL (LTL, MAL I, MAL II, PHA-L, PNA). Concentrations for analysis were determined to obtain comparable relative fluorescence units (RFU) signal above background from printed glycans on the mammalian glycan array v5.0 containing 611 glycans. FL-Spike RFUs were annotated from data in Watanabe et al. Minimum threshold for analysis was set at 5000 RFU. Force graphs and correlation mapping were projected using the GLAD online software.

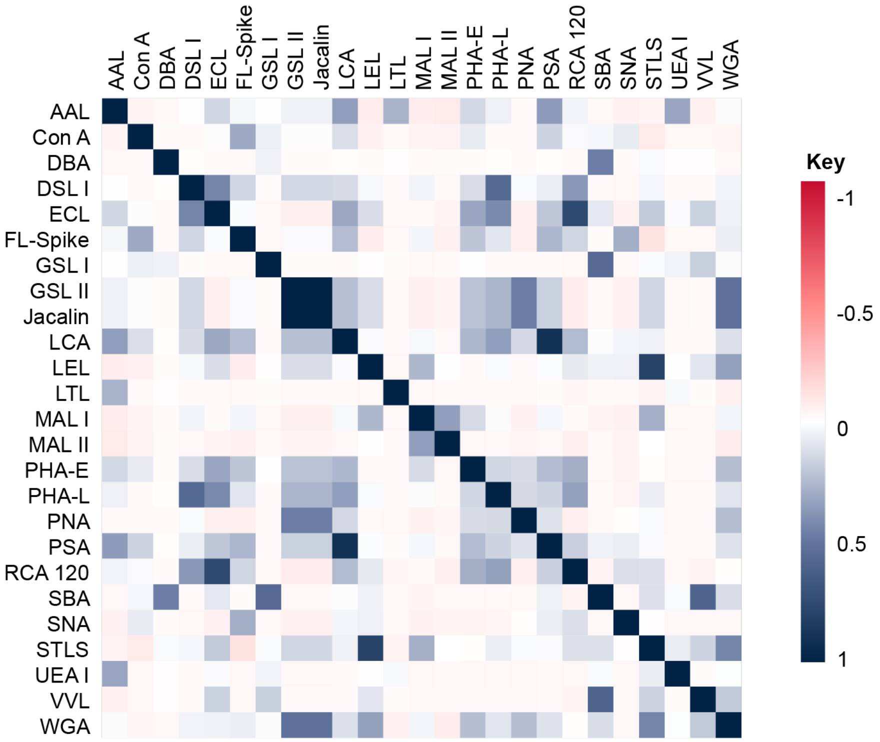

Supplementary Figure 1. Lectin correlation map of overlapping glycans from the CFG mammalian glycan array of 611 glycans plotted using the GLAD tool. FL-Spike glycan Pearson correlation analysis with lectin specificity used to predict lectin binders. Correlation ≥0.1 considered high, between 0.1 and 0 considered intermediate and negative values considered weak.

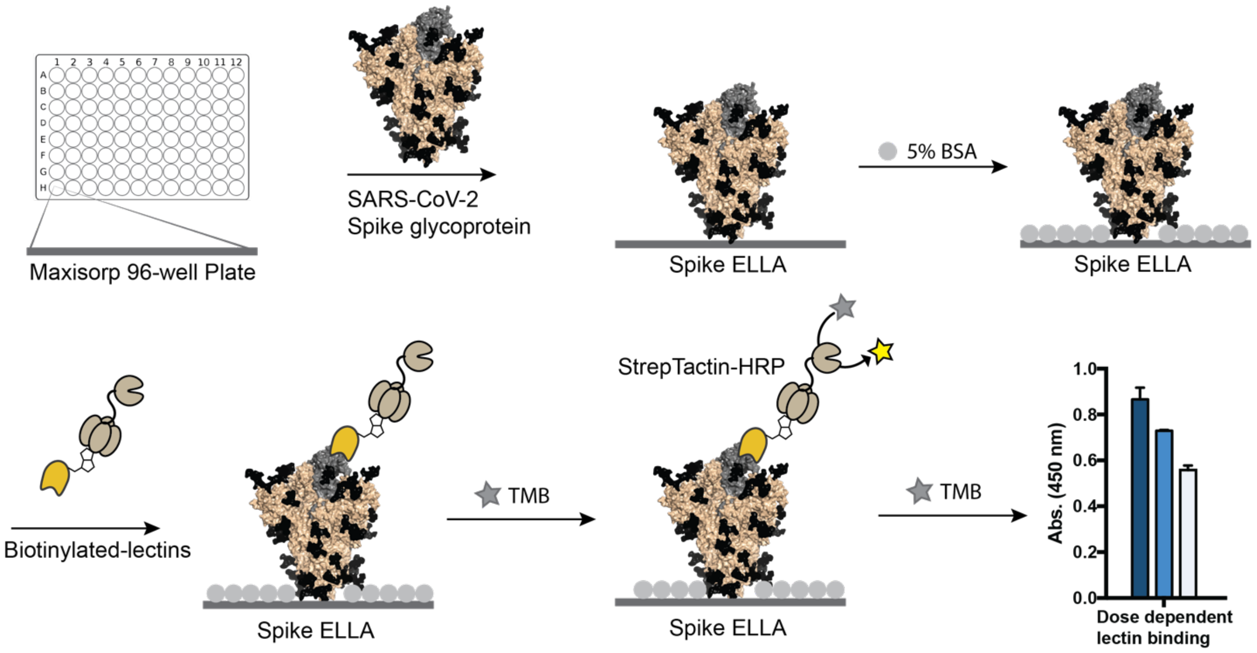

Supplementary Figure 2. Lectin fingerprinting assay. FL-Spike or RBD run against each 24 plant or 6 prokaryotic biotinylated lectins in ELLA assay to generate unique protein fingerprints.

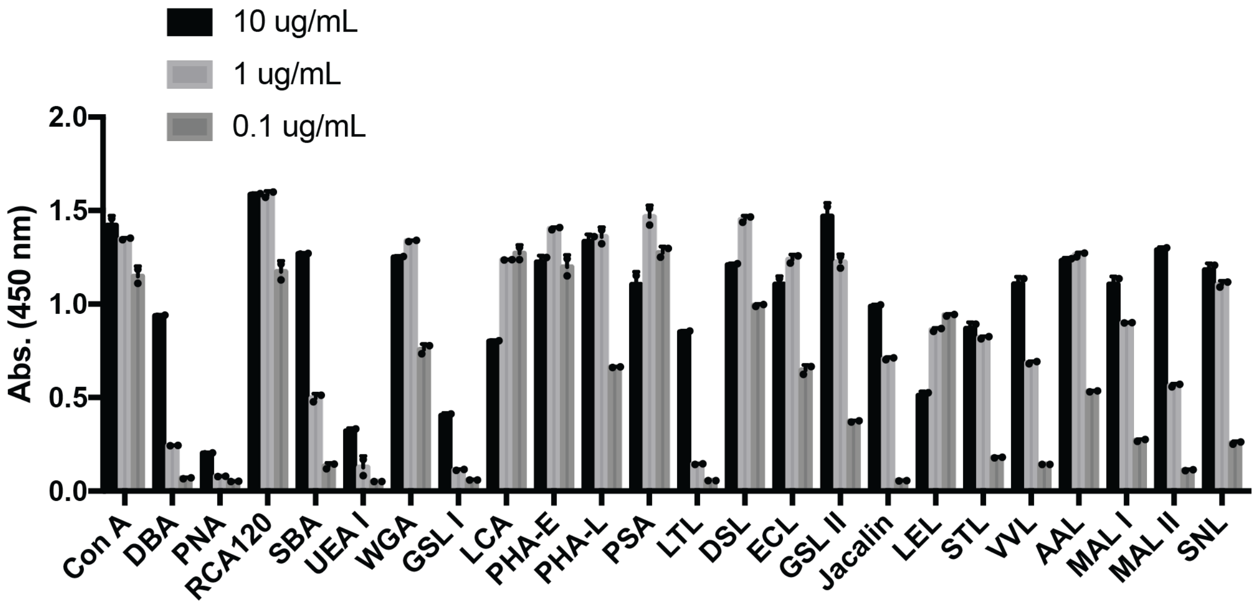

Supplementary Figure 3. Dose-dependent binding of 24 plant and fungal lectins against immobilized FL-Spike(10 μg/mL). Each lectin was added at 10, 1 and 0.1 μg/mL in duplicate and showed dose-depending binding. Fingerprints are representative of data from three individual experiments.

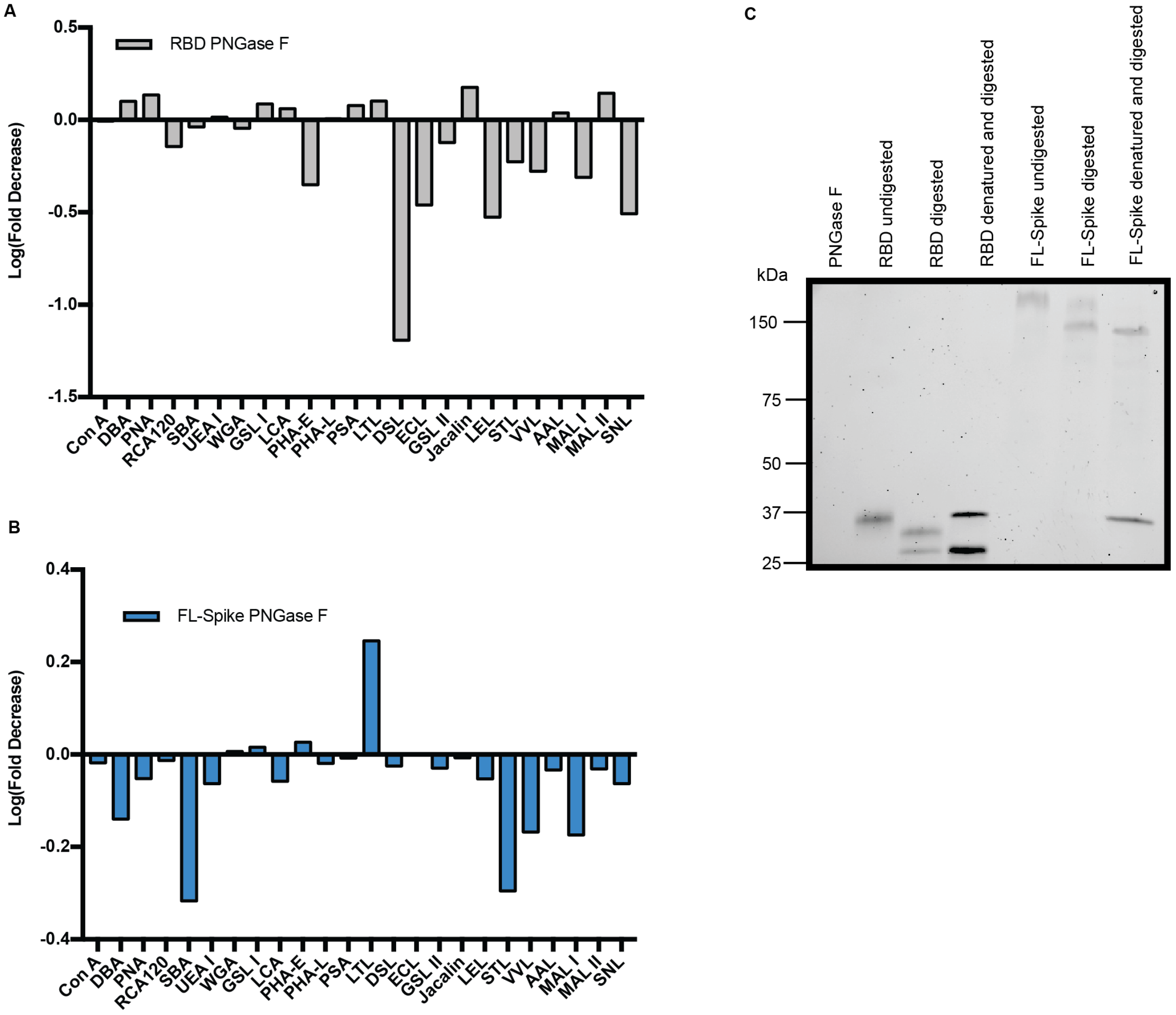

Supplementary Figure 4. PNGase F treatment of (A) RBD and (B) FL-Spike show significant reduction in lectin binding. (C) Stain-free SDS-PAGE of PNGase F treated RBD and FL-Spike does not completely remove N-glycans. Fold decrease bar graphs are representative of two individual experiments.

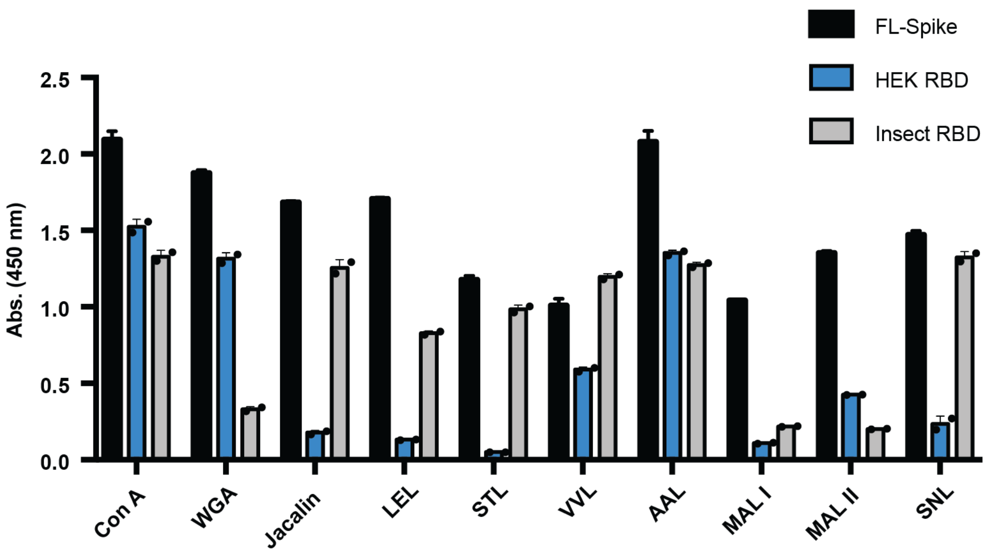

Supplementary Figure 5. Lectin fingerprints from recombinant FL-Spike and RBD from HEK and Insect cells have distinct signatures. ELLA assay with immobilized FL-Spike (black), HEK-expressed RBD (blue), or Insect-expressed RBD (grey) were treated with a panel of 10 lectins to determine differences in glycan heterogeneity. Lectin fingerprints are representative of two individual experiments.

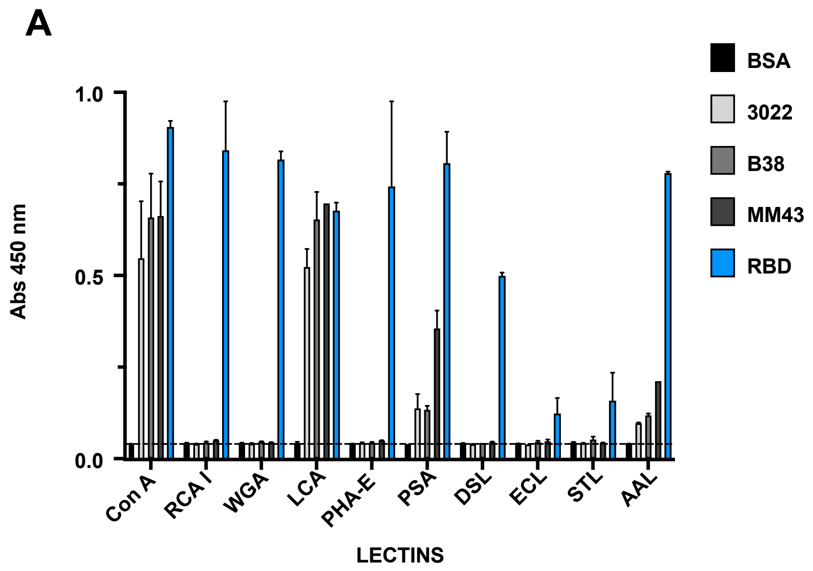

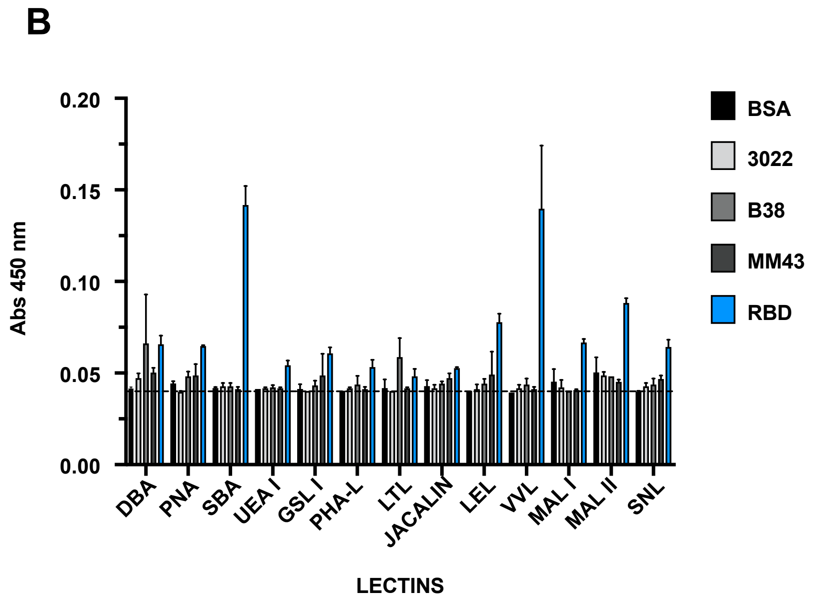

Supplementary Figure 6. Immobilized neutralizing (MM43, B38, 1 μg/mL) and non-neutralizing (CR3022, 1 μg/mL) antibodies were evaluated for binding against a panel of plant and fungal lectins (1 μg/mL) using ELISA. Antibodies were tested alongside Spike RBD (10 μg/mL) and a BSA only control (black). Dotted lines represent background binding signal threshold based on BSA controls. Data are arranged by high (A) and moderate to low (B) lectin binding to RBD. Results are representative of duplicate experiments.

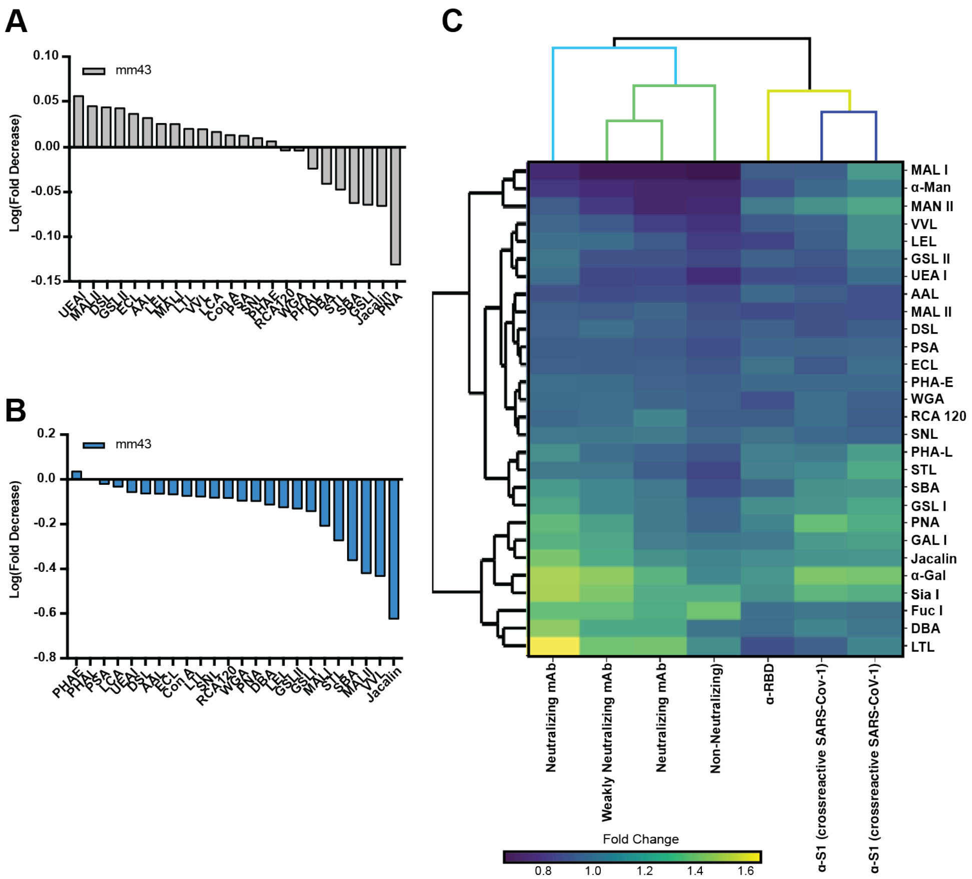

Supplementary Figure 7. Competitive lectin fingerprints indicate unique signatures for neutralizing antibodies. (A) Competitive ELLA assay showing Log (fold change) upon treatment of immobilized RBD (grey) and (B) FL-Spike (blue) containing 28 plant, fungal, and prokaryotic lectins with MM43 commercial anti-RBD antibody (1 µg/mL) in parallel. (C) Dendrogram displaying hierarchical clustering analysis of immobilized RBD with bound plant and prokaryotic lectins (1 µg/mL) treated with convalescent patient and commercial antibodies (n=7, 1 µg/mL) Dendrograms from highly potent neutralizing antibody (light blue, n=1), weakly neutralizing, non-orthosteric neutralizing, and non-neutralizing mAbs (green, n=3), commercial neutralizing mAb (yellow, n=1) and crossreactive anti-S1 (purple, n=2) antibody with SARS-CoV-1 indicate distinct and separate lectin fingerprints were observed for each sample. Heatmap plotted as a fold change of lectin signal and is representative of two experiments.

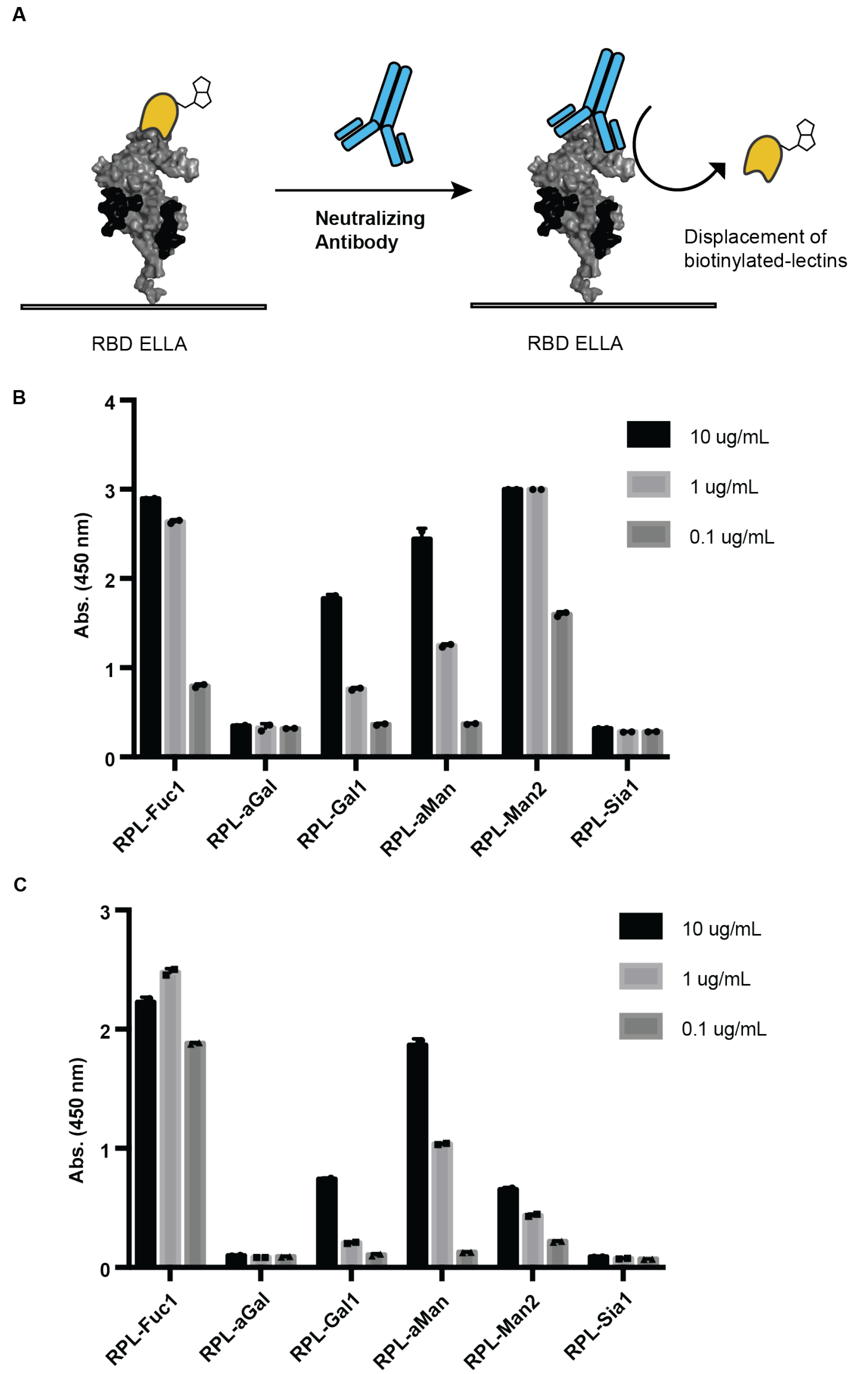

Supplementary Figure 8. Competitive lectin fingerprint assay using (A) neutralizing antibodies against immobilized RBD. Dose-dependent binding of recombinant prokaryotic lectins (RPL) at (10, 1 and 0.1 μg/mL) to (B) FL-Spike and (C) RBD. Prokaryotic lectin fingerprints shown are representative of two individual experiments.

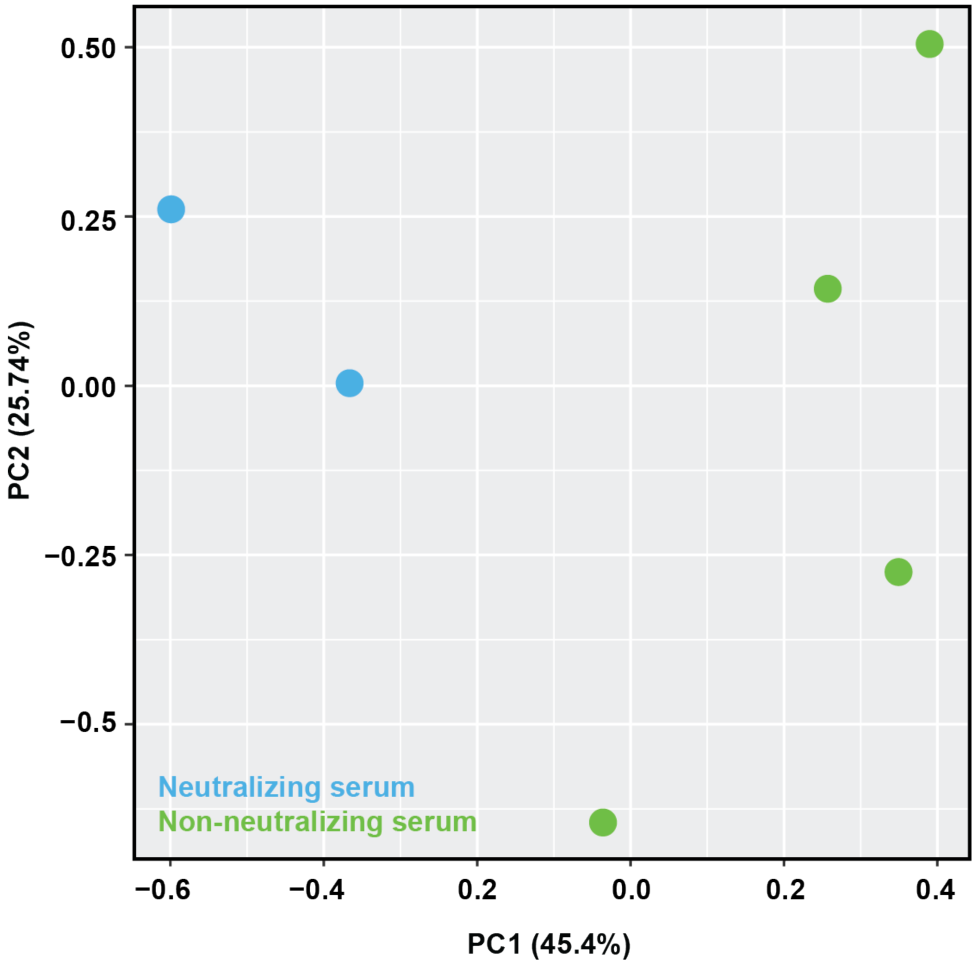

Supplementary Figure 9. Principal component analysis of lectin displacement by neutralizing (blue) and non-neutralizing (green) convalescent patient sera (1:100 dilution) on immobilized RBD. Distinct and separate lectin fingerprints were observed for each sample class. Analysis represents data from two separate experiments.

Lectin	Abbr	Origin	Specificity	Vendor	Cat. No.
Concanavalin A	ConA	plant	Mannose, Glucose	Vector Laboratories	BK-1000
Glycine max (soybean) agglutinin	SBA	plant	terminal α- or β-linked N-acetylgalactosamine and galactose	Vector Laboratories	BK-1000
Triticum vulgaris (wheat germ) agglutinin	WGA	plant	N-acetylglucosamine	Vector Laboratories	BK-1000
Dolichos biflorus agglutinin	DBA	plant	α-linked N-acetylgalactosamine	Vector Laboratories	BK-1000
Ulex europaeus agglutinin I	UEA I	plant	α-linked fucose	Vector Laboratories	BK-1000
Ricinus communis agglutinin	RCA120	plant	Galactose, Lactose	Vector Laboratories	BK-1000
Arachis hypogaea (peanut) agglutinin	PNA	plant	galactosyl (β-1,3) N-acetylgalactosamine	Vector Laboratories	BK-1000
Griffonia (Bandeiraea) simplicifolia lectin	GSL I	plant	α-N-acetylgalactosamine, α-galactose	Vector Laboratories	BK-2100
Pisum sativum lectin	PSA	plant	α-linked mannose-containing oligosaccharides, with an N-acetylchitobiose-linked α-fucose residue included	Vector Laboratories	BK-2100
Len culinaris agglutinin	LCA	plant	Mannose, Glucose	Vector Laboratories	BK-2100
Phaseolus vulgaris Erythroagglutinin	PHA-E	plant	Galactose, Complex Structures	Vector Laboratories	BK-2100
Phaseolus vulgaris Leucoagglutinin	PHA-L	plant	Galactose, Complex Structures	Vector Laboratories	BK-2100
Datura stramonium lectin	DSL	plant	(β-1,4) linked N-acetylglucosamine oligomers, N-acetyllactosamine	Vector Laboratories	BK-3000
Erythrina cristagalli lectin	ECL	plant	Galactose, N-Acetylgalactosamine, Lactose	Vector Laboratories	BK-3000
Griffonia (Bandeiraea) simplicifolia lectin II	GSL II	plant	α- or β-linked N-acetylglucosamine	Vector Laboratories	BK-3000
Jacalin	Jacalin	plant	O-glycosidically linked galactosyl (β-1,3) N-acetylgalactosamine	Vector Laboratories	BK-3000
Lycopersicon esculentum (tomato) lectin	LEL	plant	[GlcNAc]1-3, N-Acetylglucosamine	Vector Laboratories	BK-3000
Vicia villosa agglutinin	VVL	plant	α- or β-linked terminal N-acetylgalactosamine	Vector Laboratories	BK-3000
Aleuria aurantia Lectin	AAL	fungus	(α -1,2) linked fucose residues, fucose linked (α -1,6) to N-acetylglucosamine or to fucose linked (α -1,3) to N-acetyllactosamine related structures	Vector Laboratories	B-1395-1
Maackia amurensis Lectin II	MAL II	plant	sialic acid in an (α-2,3) linkage	Vector Laboratories	B-1265-1
Maackia amurensis Lectin I	MAL I	plant	gal (β-1,4) glcNAc, galactose, N-acetyllactosamine with sialic acid at the 3 position of galactose	Vector Laboratories	B-1315-2
Sambucus nigra Lectin	SNL	plant	sialic acid attached to terminal galactose in α-2,6 and to a lesser degree, α-2,3 linkage.	Vector Laboratories	B-1305-2
Lotus tetragonolobus lectin	LTL	plant	Fucose, Arabinose	Vector Laboratories	B-1325-2
Solanum tuberosum (Potato) Lectin	STL	plant	N-acetylglucosamine	Vector Laboratories	BK-3000
RPL-aGal	aGal	prokaryote	Terminal α-linked Galactose & N-Acetylgalactosamine (GalNAc)	GlycoSeLect, Ltd.	L-001
RPL-Gal1	Gal1	prokaryote	Terminal β-linked Galactose & N-Acetyllactosamine (LacNAc)	GlycoSeLect, Ltd.	L-002
RPL-aMan	aMan	prokaryote	Fucose/Mannose: Lewis a (Lea), Lewis x (Lex) & terminal α-mannose	GlycoSeLect, Ltd.	L-006
RPL-Man2	Man2	prokaryote	Terminal α -mannose	GlycoSeLect, Ltd.	L-007
RPL-Sia1	Sia1	prokaryote	Terminal α2-3-linked Sialic Acid (Neu5Ac) – on both N-linked and O-Linked Glycans	GlycoSeLect, Ltd.	L-008
RPL-Fuc1	Fuc1	prokaryote	α-linked Fucose	GlycoSeLect, Ltd.	L-011

Table S1. List of lectins used in this manuscript.

Sera	Participant ID	Description	Age	Sex	Vaccine r	Doses	Days after last dose	A (wild type)	Delta/B.1.
1	VIC-061	VIC-061 - Mod x2 (59d) [23F]	23	F	Moderna	2	59	2053	93
2	VIC-063	VIC-063 - Mod x2 (60d) [25M]	25	M	Moderna	2	60	1333	100
3	VIC-065	VIC-065 - Mod x2 (117d) [33M	33	M	Moderna	2	117	558	12
4	VIC-069	VIC-069 - Pfi x2 (2d) [28F]	28	F	Pfizer	2	2	1195	12
5	VIC-070	VIC-070 - Mod x1 (19d) [58F]	58	F	Moderna	1	19	306	12
6	VIC-201	VIC-201 - Mod x1 (21d) [28F]	28	F	Moderna	1	21	166	93
7	VIC-203	VIC-203 - Mod x1 (29d) [46F]	46	F	Moderna	1	29	26	12
8	VIC-204	VIC-204 - Pfi x2 (9d) [33F]	33	F	Pfizer	2	9	1003	485
9	VIC-205	VIC-205 - Pfi x2 (10d) [28F]	28	F	Pfizer	2	10	524	441
10	VIC-206	VIC-206 - Pfi x2 (9d) [37M]	37	M	Pfizer	2	9	621	547
11	VIC-207	VIC-207 - Pfi x2 (17d) [29F]	29	F	Pfizer	2	17	430	130
12	VIC-208	VIC-208 - Pfi x2 (17d) [29F]	29	F	Pfizer	2	17	779	417
13	VIC-209	VIC-209 - Mod x2 (108d) [29M	29	M	Moderna	2	108	557	275
14	VIC-210	VIC-210 - Mod x1 (16d) [46M]	46	M	Moderna	1	16	16	12
15	VIC-211	VIC-211 - Mod x1 (27d) [39F]	39	F	Moderna	1	27	1047	792
16	VIC-202	VIC-202 - Pfi x2 (9d) [30F]	30	F	Pfizer	2	9	343	188

Table S2. Vaccinated patient sera metadata.

References

(1) Norman, M.; Gilboa, T.; Ogata, A. F.; Maley, A. M.; Cohen, L.; Busch, E. L.; Lazarovits, R.; Mao, C.-P.; Cai, Y.; Zhang, J.; Feldman, J. E.; Hauser, B. M.; Caradonna, T. M.; Chen, B.; Schmidt, A. G.; Alter, G.; Charles, R. C.; Ryan, E. T.; Walt, D. R. Ultrasensitive High-Resolution Profiling of Early Seroconversion in Patients with COVID-19. *Nat. Biomed. Eng.* **2020**, 1–8. https://doi.org/10.1038/s41551-020-00611-x.

(2) Yang, Z.-Y.; Kong, W.-P.; Huang, Y.; Roberts, A.; Murphy, B. R.; Subbarao, K.; Nabel, G. J. A DNA Vaccine Induces SARS Coronavirus Neutralization and Protective Immunity in Mice. *Nature* **2004**, *428* (6982), 561–564. https://doi.org/10.1038/nature02463.

(3) Supekar, N. T.; Shajahan, A.; Gleinich, A. S.; Rouhani, D. S.; Heiss, C.; Chapla, D. G.; Moremen, K. W.; Azadi, P. Variable Posttranslational Modifications of Severe Acute Respiratory Syndrome Coronavirus 2 Nucleocapsid Protein. *Glycobiology* **2021**, *31* (9), 1080–1092. https://doi.org/10.1093/glycob/cwab044.

(4) Shajahan, A.; Supekar, N. T.; Gleinich, A. S.; Azadi, P. Deducing the N- and O-Glycosylation Profile of the Spike Protein of Novel Coronavirus SARS-CoV-2. *Glycobiology* **2020**. https://doi.org/10.1093/glycob/cwaa042.

(5) Natarajan, A.; Jaroentomeechai, T.; Cabrera-Sánchez, M.; Mohammed, J. C.; Cox, E. C.; Young, O.; Shajahan, A.; Vilkhovoy, M.; Vadhin, S.; Varner, J. D.; Azadi, P.; DeLisa, M. P. Engineering Orthogonal Human O-Linked Glycoprotein Biosynthesis in Bacteria. *Nat. Chem. Biol.* **2020**, *16* (10), 1062–1070. https://doi.org/10.1038/s41589-020-0595-9.

(6) Galili, T.; O’Callaghan, A.; Sidi, J.; Sievert, C. Heatmaply: An R Package for Creating Interactive Cluster Heatmaps for Online Publishing. *Bioinformatics* **2018**, *34* (9), 1600–1602. https://doi.org/10.1093/bioinformatics/btx657.
